# Systematic reviews and meta-analysis published in indexed Portuguese medical journals: time trends and critical appraisal

**DOI:** 10.1186/s12874-022-01591-z

**Published:** 2022-04-10

**Authors:** Luísa Prada, Ana Prada, Miguel Marques Antunes, Ricardo M. Fernandes, João Costa, Joaquim J. Ferreira, Daniel Caldeira

**Affiliations:** 1 Laboratory of Clinical Pharmacology and Therapeutics, Faculdade de Medicina, Universidade de Lisboa, Av. Prof. Egas Moniz, 1649-028 Lisbon, Portugal; 2 Instituto de Medicina Molecular, Faculdade de Medicina, Universidade de Lisboa, Lisbon, Portugal; 3 Instituto Politécnico de Bragança, Escola Superior de Educação, Lisbon, Portugal; 4 Serviço de Cardiologia - Centro Hospitalar Lisboa Central (CHULC), Lisbon, Portugal; 5 Centro Cardiovascular da Universidade de Lisboa - CCUL, CAML, Faculdade de Medicina, Universidade de Lisboa, Lisbon, Portugal; 6 CNS - Campus Neurológico Sénior, Torres Vedras, Portugal; 7 Serviço de Cardiologia, Hospital Universitário de Santa Maria – CHULN, Lisbon, Portugal

**Keywords:** AMSTAR-2, Quality, Systematic review, Meta-analysis, Portugal

## Abstract

**Introduction:**

Over the last years, the number of systematic reviews published is steadily increasing due to the global interest in this type of evidence synthesis. However, little is known about the characteristics of this research published in Portuguese medical journals. This study aims to evaluate the publication trends and overall quality of these systematic reviews.

**Material and methods:**

This was a methodological study. We aimed the most visible Portuguese medical journals indexed in MEDLINE. Systematic reviews were identified through an electronic search (through PUBMED). We included systematic reviews published up to August 2020. Systematic reviews selection and data extraction were done independently by three authors. The overall quality critical appraisal using the A MeaSurement Tool to Assess systematic Reviews (AMSTAR-2) was independently assessed by three authors. Disagreements were solved by consensus.

**Results:**

Sixty-six systematic reviews published in 5 Portuguese medical journals were included. Most (*n* = 53; 80.3%) were systematic reviews without meta-analysis. Up to 2010 there was a steady increase in the number of systematic reviews published, followed by a period of great variability of publication, ranging from 1 to 10 in a given year. According to the systematic reviews’ typology, most have been predominantly conducted to assess the effectiveness/efficacy of health interventions (*n* = 27; 40.9%). General and Internal Medicine (*n* = 20; 30.3%) was the most addressed field. Most systematic reviews (*n* = 46; 69.7%) were rated as being of “critically low-quality”.

**Conclusions:**

There were consistent flaws in the methodological quality report of the systematic reviews included, particularly in establishing a prior protocol and not assessing the potential impact of the risk of bias on the results.

Through the years, the number of systematic reviews published increased, yet their quality is suboptimal. There is a need to improve the reporting of systematic reviews in Portuguese medical journals, which can be achieved by better adherence to quality checklists/tools.

**Supplementary Information:**

The online version contains supplementary material available at 10.1186/s12874-022-01591-z.

## Background

Systematic reviews with or without meta-analysis play an important role in evidence-based clinical practice, since they are thought to produce high-quality evidence that help answering relevant questions in a variety of areas in healthcare. Therefore, well-conducted systematic reviews are essential to ensure transparency and to minimize biased information, providing the most valid research evidence on effects of health care interventions [1]. In the past few years, the number of publications of systematic reviews with or without meta-analysis has significantly increased [2, 3], raising concerns about their methodological quality [4, 5]. It is well documented that the reliability and validity of systematic reviews’ conclusions can be compromised by methodological flaws [6–8]. While this increasing pattern and concerns are globally known, little is known about the characteristics of this type of scientific/clinical research published in Portuguese Medical Journals.

Several tools were developed for undertaking critical appraisal of systematic reviews of healthcare interventions. Currently, one of the most widely used tool for this purpose is AMSTAR-2 (A MeaSurement Tool to Assess systematic Reviews 2). AMSTAR-2 is the update of AMSTAR, a tool designed for the critical appraisal of systematic reviews of healthcare interventions. While the original tool (AMSTAR), adapted and validated in 2007, includes 11 items [9, 10], AMSTAR-2 has 16 items in total, allowing for a more detailed assessment of the methodological quality of systematic reviews [5]. Likewise, comparing to the prior version, AMSTAR-2 also includes systematic reviews based in non-randomized studies, adding to a different scoring system that helps to reduce bias, leading to an overall score [5, 11]. Also, unlike the previous tool, AMSTAR-2 provides a detailed guidance for reviewers [5]. In general, validation studies have shown that the AMSTAR-2 tool has good measurement properties [5, 9, 12].

To understand the dynamics and patterns of this growing literature in Portugal, this study aims to evaluate the publication trend, the clinical research field, the typology of systematic reviews, and the overall methodological quality of systematic reviews (assessed using AMSTAR-2) published in indexed Portuguese medical journals.

## Methods

### Protocol registration

The protocol of this study was developed and registered in the International platform of registered systematic review and meta-analysis protocols (INPLASY) with the following registration number INPLASY202090105 (available at: https://inplasy.com/inplasy-2020-9-0105/). The protocol was not published in any peer-reviewed journal. Conduct and reporting followed the PRISMA statement [13] (see Additional file 2).

### Search methods

Potentially eligible systematic reviews with or without meta-analysis were identified through an electronic search up to August 2020, targeting Portuguese Medical journals indexed in MEDLINE, through PubMed (Search strategy at Additional file 1, Supplementary Table 2).

### Study selection

Three of the authors (LP, AP, MMA) independently screened the search results for inclusion, assessing the abstract and then the complete text, according to the inclusion and exclusion criteria. This was a methodological study [14], for which we aimed to evaluate the reporting quality of systematic reviews, with or without meta-analysis, published in the most visible Portuguese medical journals indexed in MEDLINE. Following Journal Citation Reports™ (JCR), we only included journals whose region was Portugal.

Systematic review in healthcare is a specific research design that gathers, analysis and appraises the evidence available about a specific clinical question. Systematic reviews follow a well-structured, reproducible, and transparent research methods to provide an overview of research evidence on effects of health care interventions [15]. However, there is no standard or consensus definition of this study design, which leads to a vague and ambiguous definitions of this type of research [16]. Therefore, since we aimed to assess the methodological quality of systematic reviews published in medical Portuguese journals, we decided to include reviews that included the term “systematic review” in the publication title or abstract, regardless the definition of systematic review used by the review’s authors. To increase sensitivity in our search, we also searched the term “meta-analysis” in the publication title or abstract in order to find systematic reviews with meta-analysis and minimizing the risk of losing significant studies. Also, we decided to include systematic reviews regardless of research question [17], methodological or reporting quality, and included primary studies designs.

We excluded studies which authors did not identified their study design as systematic reviews (with or without meta-analysis), systematic reviews that did not included studies enrolling human participants, as well as publications that only underwent a systematic search. Conference abstracts and letters to the editor were not included, since it was not possible to fully assess their methodological quality based on the information contained in the abstract or in the letter. We did not include Cochrane Collaboration Systematic reviews. Discrepancies were resolved by consensus-based discussion.

### Data extraction

For each of the eligible systematic reviews, three reviewers (LP, AP and MMA) independently extracted relevant data into a pre-piloted data collection template and assessed the overall quality of the systematic reviews using AMSTAR-2 tool. The specific items extracted from the full text were as follows: first author’s surname; article’s title; journal name; year of publication; type of review conducted (according to Munn et al. criteria) [17]; whether a meta-analysis has been conducted or not; country and country’s region of first author Institution, and instruments used to evaluate the risk of bias (RoB). To collect other bibliographic data, we searched the Web of Science Core Collection (WoSCC) database. WoSCC was searched by review, and we collected data, such as the clinical research field of the systematic reviews included (accessed on 10th January 2021, via https://www.webofscience.com/wos/woscc/basic-search).

### Quality assessment of methodology

AMSTAR-2 checklist aims to evaluate systematic reviews and appraises 16 domains (for detail, visit https://amstar.ca/Amstar_Checklist.php). The possible answers for these domains include: “Yes”, “Partial Yes”, “No” or “No meta-analysis conducted”. If the item was answered correctly and well-documented, the judgment was “Yes”; if the item was answered correctly with limited evidence, the answer was “Partial Yes”; if no information was provided to rate the item, the judgment was “No”. Seven of the 16 domains are critical, including prior protocol, comprehensive literature search, justification of excluding studies, assessment of RoB for individual studies, appropriate meta-analytic methods, consideration of RoB in results and impact of publication bias (items 2, 4, 7, 9, 11, 13 and 15). Item 11 and 15 are only assessed if a meta-analysis was performed. Based on weaknesses in critical domains, the overall confidence in the results of Systematic reviews and meta-analysis can be divided into: as “High” (none/one non-critical weakness), “Moderate” (> 1 non-critical weakness), “Low” (one critical flaw) and “Critically Low” (> 1 critical flaw). The methodological quality assessment of the systematic reviews and meta-analysis was independently assessed by two reviewers in pairs (LP and AP, MMA and LP) using the AMSTAR-2 tool. All reviewers had undergone methodological quality assessment calibration exercises. Discrepancies were resolved by consensus-based discussion.

### Data analysis

The collected data were entered and checked in a pre-piloted form. The descriptive statistical analyses included calculations of absolute and relative frequencies of the qualitative variables.

## Results

### General characteristics and temporal trends

**Fig. 1:**
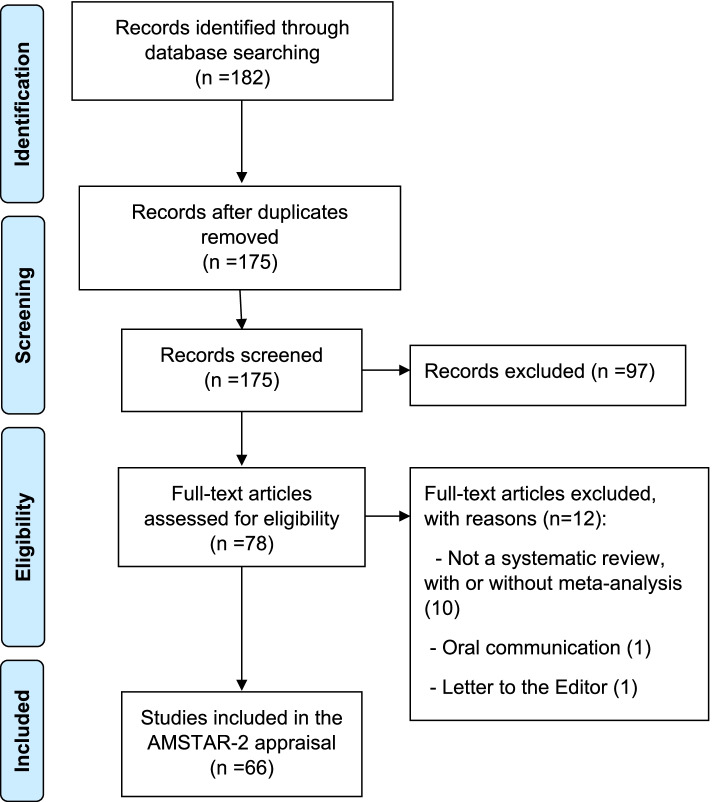
Flowchart showing the search results and reasons for exclusion

We identified and assessed sixty-six systematic reviews published between 2001 and August 2020 (The flowchart in Fig. 1 illustrates the selection process).

We identified 5 Portuguese medical journals indexed to MEDLINE. Table 1 and Supplementary Table 3 (Additional file 1) summarises the general characteristics of the systematic reviews included. Twenty one (31.8%) of systematic reviews included were published in *Acta Médica Portuguesa*, 16 (24.2%) in *Acta Reumatológica Portuguesa*, 14 (21.2%) in Pulmonology (previously Revista Portuguesa de Pneumologia), 13 (19.7%) in *Revista Portuguesa de Cardiologia*, and 2 (3.0%) in *Revista Portuguesa de Cirurgia Cardio-torácica e Vascular*. Systematic reviews without meta-analysis were more frequently published (*n* = 53; 80.3%) in comparison to systematic reviews with meta-analysis (*n* = 13; 19.7%).

**Table 1:** General characteristics of Systematic reviews included

**Main clinical research field targeted in the included systematic reviews, n (%)**	
General and Internal Medicine	20 (30.3%)
Rheumatology	16 (24.2%)
Respiratory System	13 (19.7%)
Cardiovascular System and Cardiology	11 (16.7%)
Pathology	1 (1.5%)
Pharmacology and Pharmacy	1 (1.5%)
Psychiatry	1 (1.5%)
Transplantation	1 (1.5%)
NA	2 (3.0%)
**Type of systematic reviews included according to Munn et al.** [17] **criteria, n (%)**	
Effectiveness/Efficacy	27 (40.9%)
Expert opinion or policy	11 (16.7%)
Prognostic	10 (15.2%)
Prevalence	8 (12.1%)
Diagnostic Test Accuracy	6 (9.1%)
Costs/Economic evaluation	2 (3.0%)
Etiology or risk	1 (1.5%)
Experimental (qualitative)	1 (1.5%)
**Country Region of the Institution of the Portuguese Systematic reviews’ first author, n (%)**	
Portugal	49 (74.2%)
Brazil	11 (16.7%)
Italy	3 (4.5%)
China	1 (1.5%)
Spain	1 (1.5%)
USA	1 (1.5%)
**Region of Portugal from the institution of the first author, n (%)**^a^	
North	25 (51.0%)
Lisbon and Tagus Valley	17 (34.7%)
Center	7 (14.3%)
Alentejo	0 (0%)
Algarve	0 (0%)
Azores	0 (0%)
Madeira	0 (0%)
**Distribution of the included systematic reviews according to the journal of publication, n (%)**	
*Acta Médica Portuguesa*	21 (31.8%)
*Acta Reumatológica Portuguesa*	16 (24.2%)
Pulmonology (previously *Revista Portuguesa de Pneumologia*)	14 (21.2%)
*Revista Portuguesa de Cardiologia*	13 (19.7%)
*Revista Portuguesa de Cirurgia Cardio-torácica e Vascular*	2 (3.0%)
**Publication year, median (range)**	2014 (2001–2020)
**Publication journal impact factor, median (range)**^b^	1.3 (1.1–3.6) ^c^
**Included a PRISMA-like flow diagram, n (%)**	37 (56.1%)
**Number of review authors, median (range)**	4 (1–12)
**Systematic reviews with only one author, n (%)**	2 (3.0%)
**Eligibility criteria based on language of publication, n (%)**	
English and non-English	31 (47.0%)
Not reported	18 (27.3%)
English publications only	17 (25.8%)
**Eligibility of study design, n (%)**	
Only observational studies	28 (42.4%)
Only RCT’s studies	12 (18.2%)
RCT’s and observational studies	12 (18.2%)
Not reported	12 (18.2%)
RCT’s, observational studies and reviews	1 (1.5%)
Observational studies and reviews	1 (1.5%)
**Assessing quality/methodology of primary studies, n (%)**	
Not reported	38 (57.6%)
PEDro scale	7 (10.6%)
Cochrane risk-of-bias tool	6 (9.1%)
Custom scale	5 (7.6%)
SORT scale	3 (4.5%)
MINORS tool	2 (3.0%)
OCEBM Levels of Evidence	2 (3.0%)
QUADAS tool	1 (1.5%)
QUADAS-2 tool	1 (1.5%)
MORE checklist	1 (1.5%)

*NA* Unclear, *RCT’s* Randomized controlled trials, *MORE* Methodological Evaluation of Observational Research, *OCEBM* Oxford Centre for Evidence-Based Medicine, *SORT* Strength of Recommendation Taxonomy

^a^ n (Portugal) = 49

^b^ Journal Citation Reports (JCR), Clarivate Analytics

^c^*Revista Portuguesa de Cirurgia Cardio-torácica e Vascularis* is not indexed in the JCR database

The most frequent research field covered by systematic reviews was General and Internal Medicine (30.3%). Two articles [18, 19] (3.0%) did not have their research category/ classification available in WoSCC database. Following the Munn et al. [17] criteria, 27 systematic reviews (40.9%) were classified as “effectiveness/efficacy”, 11 (16.7%) as “expert opinion or policy”, 10 (15.2%) as “prognostic”, 8 (12.1%) as “prevalence” and 6 (9.1%) as “diagnostic test accuracy”. Very few systematic reviews were categorized as being “costs/economic evaluation”, “etiology or risk” or “experimental (qualitative)”. According to the country of the first author’s institution, the majority were from Portugal (74.2%).

**Fig. 2:**
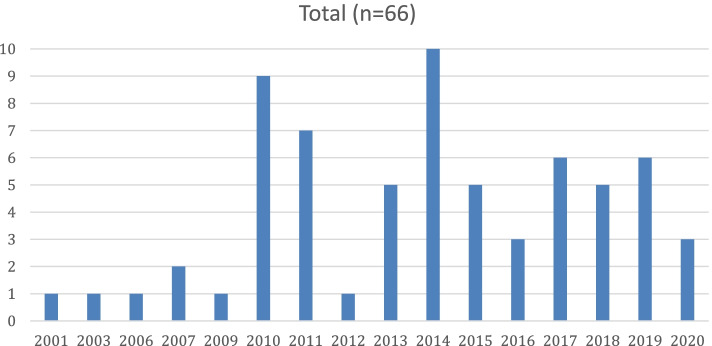
Number of reviews published per year

Prior to 2010, no more than two systematic reviews were published per year. There was a peak in publications in 2014 (*n* = 10) and since then, while the trend has been variable, no less than three systematic reviews were published per year (Fig 2).

### Methodological quality

The overall confidence in the results of 46 systematic reviews (69.7%) were rated as “Critically Low quality review”, 14 (21.2%) were rated as “Low quality” and 4 (6.1%) were rated as “Moderate quality” and only two (3.0%) were rated as “High quality”. The critical items of AMSTAR-2 mostly missed in the reporting of systematic reviews were: 48 reviews (72.7%) did not adhere to a priory well-designed protocol (question 2); 48 reviews (72.7%) partially reported only some features of a comprehensive literature search (question 4); none of the systematic reviews reported a complete description of the methods used to search for relevant literature; most systematic reviews (60.6%) did not assess RoB in individual studies that were included in the review (question 9) nor discussed the RoB (68.2%) in the results interpretation (questions 13). Nevertheless, among the 13 systematic reviews that performed a meta-analysis, all reviews used appropriate methods for the statistical combination of results (question 11) and 10 of the 13 systematic reviews with meta-analysis (76.9%) conducted a publication bias analysis and discussed its impact (question 15) (see Additional file 1, Supplementary Fig. 1 and Supplementary Table 1 for quality appraisal of systematic reviews using AMSTAR-2).

**Fig. 3:**
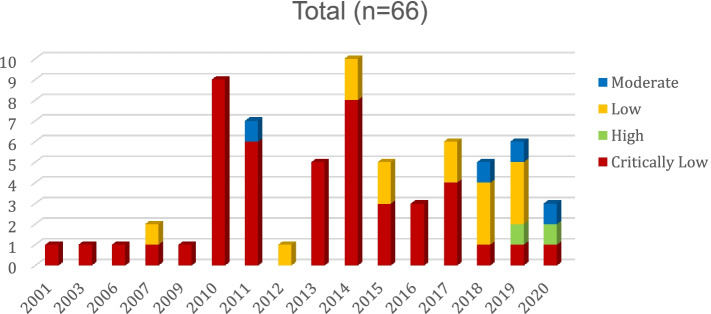
Overall methodological quality score of systematic reviews published up to August 2020

The methodological quality of the published systematic reviews has improved over time (Fig. 3), showing a decrease of “Critically Low quality” studies, however the low number of published systematic reviews precludes a robust evaluation.

## Discussion

To the best of our knowledge, our study is the first to investigate the general characteristics and assess the methodological quality using AMSTAR-2 tool of systematic reviews with or without meta-analysis published in Portuguese Medical journals indexed in MEDLINE.

The main findings were: the peak of publications of systematic reviews was in 2014 and there was no substantial increase in publications during the last decade; The majority of the systematic reviews was classified as being of “Critically Low” quality according to the AMSTAR-2 tool. Nonetheless, their overall quality has probably increased overtime.

According to our results, while there was a slight increase in the number of systematic reviews publications in the last decade comparing to the prior decade, we did not find it to be a sustained one. This trend was not expectable since systematic reviews are being increasingly published in medical journals globally [2, 20]. The potential explanatory reasons include editorial policies or authors’ choices.

Most of the included systematic reviews were assessed as being of “critically low” overall quality, which is a matter of concern. Few systematic reviews adequately satisfied critical items such as the use of a prior protocol and the assessment of RoB. Establishing a prior well-developed protocol before commencement of the review can reduce the risk of bias and promote transparency in the review process [21, 22], ideally through platforms such as International prospective register of systematic reviews (PROSPERO) or the International platform of registered systematic review and meta-analysis protocols (INPLASY). It is also important to highlight that none of the included reviews did a complete description of the methods used to search for relevant literature. This disables the reproducibility of the search, which is one of the crucial foundations for the process of systematic reviews. Also, a complete and transparent search process is the best way to avoid publication bias [15]. The absence of a clear description about the details of the literature search strategy is an important flaw. Another concern raised by our analysis is that most of the included studies did not assessed the potential impact of RoB on the results, which is essential to evaluate the validity of the SR results [15]. Furthermore, some authors failed to assess RoB with validated tools, as they developed and used their own tools to do so [23, 24].

Other studies have assessed the methodological quality of systematic reviews, with or without meta-analysis, published in healthcare/medical journals, using AMSTAR-2 tool. Most studies report similar results, with a large number of systematic reviews included being rated as low or critical low quality [25–29]. Overall, studies report that methodological and reporting quality of systematic reviews were low. There is an urgent need to address this issue since systematic reviews play a crucial role in informing patient care, policies, and decisions around populational health. This is a call for attention and action for multiple stakeholders, like journal editors and peer reviewers. Efforts must be made to maximize the transparency and completeness of reporting.

Currently, there are some quality appraisal tools and guidelines that might help authors and peer reviewers to improve and assess methodological report and review of systematic reviews. The AMSTAR-2 is one of these tools. We used this tool in our study because it allows to categorize systematic reviews [5, 9]. There are other tools, such as PRISMA [13] or MOOSE [30], which are also extensive tools/checklists that inform and help to improve the quality of the reporting of medical research, but do not have critical items nor categorize the systematic reviews according to their reporting.

We acknowledge that most of the reviews here included are not contemporary of AMSTAR-2 (nor other instruments that could improve reporting such as PRISMA). However, we would like to claim that the methodological standards for publication in Portuguese medical journals should be high and similar to other European and North American journals. We would like to stress the need for editorial policy measures for authors, editors and peer reviewers, like adherence to checklist items and prospective registration of protocols for systematic reviews, in order to strengthen the methodological quality and reliability of systematic reviews with or without meta-analysis published in Portuguese medical journals [31]. As an example, there are journals that require the submission of a methodological checklist filled by authors for editors/reviewer assessment (such as PRISMA), while others started to apply AMSTAR-2 to screen systematic reviews’ methodological quality, in order to promote reviews of higher quality [32].

We also would like to acknowledge that AMSTAR-II has limitations because it is a tool for critical appraisal mostly adapted for interventions/exposures. Also, we were conservative (with less criticism in the evaluation) when appraising non-interventional systematic reviews. The authors of AMSTAR-2 do not recommend an overall score, despite the electronic version calculates a final score [5]. In fact, we did not use the score directly, instead we used their categorization which we found useful to highlight that the overall quality is low and that publication requirements should be more demanding. Lastly, some of the included reviews, which were classified as being “critically low quality reviews”, fail to follow crucial methodological steps in conducting a qualitative or quantitative systematic reviews, flaws that call into question its classification as systematic reviews. Given the pedagogical intent and the aim of our study, we opted to include them in the final analysis.

## Conclusion

In the last decade there was an increase of published systematic reviews in indexed Portuguese Medical Journals. Regarding the methodological quality and reporting, most were evaluated as being of “Critically Low quality” according to the AMSTAR-2, mainly due to the absence of protocol registration and adequate RoB when interpreting/discussing the results of the review. This call out for a need to improve the reporting of systematic reviews, which can be made by better adherence to quality checklists/tools. Furthermore, specific quality control at the level of journal editors and peer reviewers is warrant, like implementation and adherence to checklist items in order to improve methodological quality of systematic reviews with or without meta-analysis published in Portuguese medical journals.

## Supplementary Information

**Additional file 1: Supplementary Figure 1**. Distribution (%) of AMSTAR-2 items in the systematic reviews following assessment. **Supplementary Table 1**. Assessment of the individual items of AMSTAR-2. **Supplementary Table 2**. Search Strategy. **Supplementary Table 3**. General characteristics and PICO framework (PRISMA 2020) of SRs included.

**Additional file 2.** PRISMA Checklist-Systematic reviews and meta-analyses published in indexed Portuguese Medical journals: time trends and critical appraisal.

## Data Availability

The datasets used or analysed during the current study available from the corresponding author on reasonable request.
