# Supplementary material for: Systematic reviews and meta-analysis published in indexed Portuguese medical journals: time trends and critical appraisal
Source: BMC Med Res Methodol. 2022 Apr 10;22:105. doi: 10.1186/s12874-022-01591-z (PMC8996638; doi:10.1186/s12874-022-01591-z)
Supplement: Supplementary file 1 — Additional file 1: Supplementary Figure 1. Distribution (%) of AMSTAR-2 items in the systematic reviews following assessment. Supplementary Table 1. Assessment of the individual items of AMSTAR-2. Supplementary Table 2. Search Strategy. Supplementary Table 3. General characteristics and PICO framework (PRISMA 2020) of SRs included. [file 12874_2022_1591_MOESM1_ESM.docx]

**SUPPLEMENTARY DATA**

**Overview of systematic reviews and meta-analysis published in Portuguese Medical journals: time trends and critical appraisal.**

Luísa Prada MD, Ana Prada PhD, Miguel Marques Antunes MD, Ricardo Fernandes MD PhD, João Costa MD PhD, Joaquim J. Ferreira MD PhD, Daniel Caldeira MD PhD

**Index**

[Supplementary figure 1 – Distribution (%) of AMSTAR-2 items in the systematic reviews following assessment. 3](#_Toc99011045)

[Supplementary table 1 - Assessment of the individual items of AMSTAR-2 4](#_Toc99011046)

[Supplementary table 2 – Search Strategy 11](#_Toc99011047)

[Supplementary table 3 - General characteristics and PICO framework (PRISMA 2020^(72)^) of SRs included 12](#_Toc99011048)

Supplementary figure 1 – Distribution (%) of AMSTAR-2 items in the systematic reviews following assessment.

Overall AMSTAR-II items (n=66)

* items that did not apply to all studies (only meta-analysis) and the denominator is different (n = 13)

Supplementary table 1 - Assessment of the individual items of AMSTAR-2

| **Author, Publication Year** | **Portuguese Medical Journal** | **Overall rating** | **item**  **1** | **Item**  **2†** | **Item**  **3** | **Item**  **4†** | **Item**  **5** | **Item**  **6** | **Item**  **7†** | **Item**  **8** | **Item**  **9†** | **Item**  **10** | **Item**  **11†** | **Item**  **12** | **Item**  **13†** | **Item**  **14** | **Item**  **15†** | **Item**  **16** |
| --- | --- | --- | --- | --- | --- | --- | --- | --- | --- | --- | --- | --- | --- | --- | --- | --- | --- | --- |
| AbuNurah *et al.*  2020 (1) | Pulmonology | Moderate quality review | Yes | Yes | Yes | Partial yes | Yes | Yes | Partial yes | Partial yes | Yes | No | No MA | No MA | Yes | Yes | No MA | Yes |
| Alarcão *et al.*  2013 (2) | Acta Médica Portuguesa | Critically Low quality review | Yes | No | Yes | Partial yes | No | Yes | No | Partial yes | No | No | No MA | No MA | No | No | No MA | Yes |
| Albuquerque Baltar *et al.*  2010 (3) | Pulmonology | Critically Low quality review | Yes | No | No | Partial yes | No | No | No | Partial yes | No | No | No MA | No MA | No | Yes | No MA | No |
| Almeida *et al.*  2020 (4) | Acta Médica Portuguesa | Critically Low quality review | Yes | No | No | Partial yes | No | No | No | Partial yes | Yes | No | No MA | No MA | Yes | Yes | No MA | Yes |
| Andrade da Cunha *et al.*  2010 (5) | Pulmonology | Critically Low quality review | Yes | No | No | Partial yes | Yes | No | No | Partial yes | No | No | No MA | No MA | No | Yes | No MA | No |
| Andrade *et al.*  2017 (6) | Acta Médica Portuguesa | Critically Low quality review | Yes | Partial yes | No | Partial yes | Yes | Yes | Yes | Partial yes | No | No | No MA | No MA | No | No | No MA | Yes |
| Bogas *et al.*  2011 (7) | Acta Reumatológica Portuguesa | Critically Low quality review | Yes | No | No | No | No | No | No | No | No | No | No MA | No MA | No | No | No MA | Yes |
| Branco *et al.*  2009 (8) | Acta Reumatológica Portuguesa | Critically Low quality review | No | No | No | No | No | No | No | No | No | No | No MA | No MA | No | No | No MA | No |
| Caldeira *et al. **  2014 (9) | Revista Portuguesa de Cardiologia | Critically Low quality review | Yes | No | Yes | Partial yes | Yes | Yes | Partial yes | Partial yes | Yes | No | Yes | Yes | Yes | Yes | No | Yes |
| Caldeira *et al. **  2014a (10) | Revista Portuguesa de Cardiologia | Low quality review | Yes | No | Yes | Partial yes | Yes | Yes | Partial yes | Yes | Yes | No | Yes | Yes | Yes | Yes | Yes | Yes |
| Campanilho-Marques *et al.*  2014 (11) | Acta Reumatológica Portuguesa | Critically Low quality review | Yes | No | No | No | No | No | Yes | Yes | No | No | No MA | No MA | No | No | No MA | No |
| Carmona *et al.*  2007 (12) | Acta Reumatológica Portuguesa | Critically Low quality review | Yes | No | No | Partial yes | No | No | No | No | No | No | No MA | No MA | No | No | No MA | No |
| Cavaca *et al.*  2017 (13) | Revista Portuguesa de Cardiologia | Critically Low quality review | Yes | No | No | No | No | No | Yes | Partial yes | Yes | No | No MA | No MA | No | No | No MA | Yes |
| Cavalcante *et al. **  2019 (14) | Revista Portuguesa de Cardiologia | High quality review | Yes | Partial yes | Yes | Partial yes | Yes | Yes | Yes | Yes | Yes | No | Yes | Yes | No | Yes | Yes | Yes |
| Cavalcante Marcelino *et al.*  2010 (15) | Pulmonology | Critically Low quality review | Yes | No | No | Partial yes | Yes | Yes | Yes | Partial yes | No | No | No MA | No MA | No | No | No MA | No |
| Coelho *et al.*  2010 (16) | Acta Médica Portuguesa | Critically Low quality review | No | No | No | Partial yes | No | No | No | No | No | No | No MA | No MA | No | No | No MA | Yes |
| Costa *et al.*  2003 (17-19) | Revista Portuguesa de Cardiologia | Critically Low quality review | Yes | No | No | No | Yes | Yes | No | Partial yes | No | No | No MA | No MA | No | Yes | No MA | No |
| Costa *et al.*  2001 (20-23) | Revista Portuguesa de Cardiologia | Critically Low quality review | Yes | No | No | Partial yes | Yes | Yes | No | No | No | No | No MA | No MA | No | No | No MA | No |
| Costa *et al.*  2018a (24) | Acta Médica Portuguesa | Moderate quality review | Yes | Partial yes | No | Partial yes | Yes | Yes | Partial yes | Partial yes | Yes | No | No MA | No MA | Yes | Yes | No MA | Yes |
| Costa *et al.*  2018 (25) | Acta Reumatológica Portuguesa | Critically Low quality review | Yes | Partial yes | No | Partial yes | Yes | Yes | Partial yes | Yes | No | No | No MA | No MA | No | No | No MA | No |
| Couto *et al. **  2014 (26) | Acta Médica Portuguesa | Critically Low quality review | Yes | No | No | Partial yes | Yes | Yes | Partial yes | No | No | No | Yes | No | No | Yes | Yes | Yes |
| Dores *et al.*  2013 (27) | Acta Médica Portuguesa | Critically Low quality review | Yes | No | No | No | Yes | Yes | No | No | No | No | No MA | No MA | No | No | No MA | Yes |
| Fabiao *et al.*  2010 (28) | Acta Médica Portuguesa | Critically Low quality review | Yes | No | No | No | No | No | No | Partial yes | No | No | No MA | No MA | No | No | No MA | Yes |
| Feitosa *et al.*  2012 (29) | Pulmonology | Low quality review | Yes | No | No | Partial yes | Yes | Yes | Partial yes | Partial yes | Yes | No | No MA | No MA | Yes | Yes | No MA | Yes |
| Ferreira *et al.*  2015 (30) | Revista Portuguesa de Cardiologia | Low quality review | Yes | No | No | Partial yes | No | No | Partial yes | Partial yes | Yes | No | No MA | No MA | No | No | No MA | Yes |
| Ferreira *et al.*  2011 (31) | Acta Médica Portuguesa | Critically Low quality review | Yes | No | No | Partial yes | No | No | No | Partial yes | No | No | No MA | No MA | No | No | No MA | Yes |
| Ferreira *et al. **  2019 (32) | Acta Reumatológica Portuguesa | Low quality review | Yes | Partial yes | Yes | Partial yes | Yes | Yes | Partial yes | Yes | Yes | No | Yes | Yes | Yes | Yes | No | Yes |
| Fölter *et al.*  2011 (33) | Acta Reumatológica Portuguesa | Critically Low quality review | Yes | No | No | No | No | No | No | Partial yes | No | No | No MA | No MA | No | No | No MA | No |
| Francica *et al.*  2014 (34) | Acta Médica Portuguesa | Critically Low quality review | Yes | No | No | Partial yes | Yes | Yes | Yes | Partial yes | No | No | No MA | No MA | No | No | No MA | Yes |
| Gomes Dos Reis Pimentel *et al.*  2017 (35) | Pulmonology | Low quality review | Yes | Yes | Yes | No | No | No | Partial yes | Partial yes | Yes | No | No MA | No MA | Yes | Yes | No MA | Yes |
| Gouveia *et al.*  2018 (36) | Acta Médica Portuguesa | Low quality review | Yes | No | Yes | Partial yes | No | No | Partial yes | Yes | Yes | No | No MA | No MA | Yes | Yes | No MA | Yes |
| Guedes *et al.*  2014 (37) | Revista Portuguesa de Cardiologia | Critically Low quality review | Yes | No | No | Partial yes | No | No | No | Yes | No | No | No MA | No MA | No | Yes | No MA | Yes |
| Guerreiro *et al.*  2011 (38) | Acta Médica Portuguesa | Critically Low quality review | No | No | No | No | No | No | No | No | No | No | No MA | No MA | No | No | No MA | Yes |
| Ladeiras-Lopes *et al. **  2019 (39) | Revista Portuguesa de Cardiologia | Low quality review | Yes | Partial yes | Yes | No | No | Yes | Partial yes | Yes | Yes | No | Yes | Yes | Yes | Yes | Yes | Yes |
| Liz Almeida *et al.*  2015 (40) | Revista Portuguesa de Cardiologia | Critically Low quality review | Yes | No | No | No | No | No | Partial yes | No | No | No | No MA | No MA | No | No | No MA | Yes |
| Lopes *et al.*  2016 (41) | Acta Reumatológica Portuguesa | Critically Low quality review | Yes | No | No | No | Yes | Yes | Yes | Yes | Yes | No | No MA | No MA | Yes | No | No MA | No |
| Lopes *et al. **  2014 (42) | Revista Portuguesa de Cardiologia | Critically Low quality review | Yes | No | No | Partial yes | No | No | Yes | Partial yes | No | No | Yes | No | No | Yes | Yes | Yes |
| Lopes-Conceição *et al.*  2014 (43) | Revista Portuguesa de Cardiologia | Critically Low quality review | Yes | No | No | No | Yes | Yes | Yes | No | No | No | No MA | No MA | No | Yes | No MA | Yes |
| Mancuso *et al.*  2006 (44) | Pulmonology | Critically Low quality review | Yes | No | No | Partial yes | No | No | No | No | No | No | No MA | No MA | No | No | No MA | No |
| Mancuzo *et al.*  2010 (45) | Pulmonology | Critically Low quality review | Yes | No | No | Partial yes | No | No | No | No | No | No | No MA | No MA | No | No | No MA | No |
| Marinho-Dias *et al.*  2013 (46) | Acta Médica Portuguesa | Critically Low quality review | Yes | No | No | No | No | No | Partial yes | No | No | No | No MA | No MA | No | No | No MA | Yes |
| Martins *et al. **  2014 (47) | Acta Reumatológica Portuguesa | Low quality review | Yes | Partial yes | Yes | Partial yes | Yes | Yes | Partial yes | Partial yes | Yes | No | Yes | Yes | Yes | Yes | No | No |
| Melo da Silva *et al. **  2015 (48) | Revista Portuguesa de Cirurgia Cardio-torácica e Vascular | Low quality review | Yes | No | Yes | Partial yes | Yes | Yes | Partial yes | Partial yes | Yes | No | Yes | Yes | Yes | Yes | Yes | No |
| Monjardino *et al.*  2011 (49) | Acta Reumatológica Portuguesa | Moderate quality review | Yes | Partial yes | Yes | Partial yes | Yes | Yes | Yes | No | Yes | No | No MA | No MA | Yes | Yes | No MA | No |
| Pacheco de Oliveira *et al.*  2015 (50) | Revista Portuguesa de Cirurgia Cardio-torácica e Vascular | Critically Low quality review | Yes | Partial yes | No | No | No | No | No | No | No | No | No MA | No MA | No | No | No MA | No |
| Padilha J.M.  2010 (51) | Pulmonology | Critically Low quality review | Yes | No | No | Partial yes | No | No | No | Partial yes | No | No | No MA | No MA | No | No | No MA | No |
| Paneroni *et al. **  2020 (52) | Pulmonology | High quality review | Yes | Partial yes | Yes | Partial yes | Yes | Yes | Partial yes | Yes | Yes | No | Yes | Yes | Yes | Yes | Yes | Yes |
| Peixoto *et al.*  2013 (53) | Acta Reumatológica Portuguesa | Critically Low quality review | Yes | No | No | Partial yes | No | No | No | Partial yes | No | No | No MA | No MA | No | No | No MA | No |
| Pereira Da Silva *et al.*  2010 (54) | Acta Médica Portuguesa | Critically Low quality review | Yes | No | No | Partial yes | No | No | No | Partial yes | No | No | No MA | No MA | No | No | No MA | Yes |
| Pinheiro *et al. **  2019 (55) | Acta Médica Portuguesa | Moderate quality review | Yes | Partial yes | Yes | Partial yes | No | No | Partial yes | No | Yes | No | Yes | Yes | Yes | Yes | Yes | Yes |
| Pisani *et al.*  2019 (56) | Pulmonology | Critically Low quality review | Yes | No | No | Partial yes | Yes | Yes | Partial yes | No | Yes | No | No MA | No MA | No | No | No MA | No |
| Quintas-Neves *et al.*  2016 (57) | Pulmonology | Critically Low quality review | Yes | No | Yes | No | No | No | No | No | No | No | No MA | No MA | No | No | No MA | Yes |
| Raposo *et al.*  2016 (58) | Acta Reumatológica Portuguesa | Critically Low quality review | Yes | Partial yes | No | No | No | No | Yes | Yes | No | No | No MA | No MA | No | No | No MA | No |
| Ribeiro *et al.*  2010 (59) | Acta Reumatológica Portuguesa | Critically Low quality review | Yes | Partial yes | No | Partial yes | No | No | No | No | Yes | No | No MA | No MA | No | Yes | No MA | No |
| Ribeiro *et al.*  2007 (60) | Acta Reumatológica Portuguesa | Low quality review | Yes | No | Yes | Partial yes | Yes | Yes | Partial yes | No | Yes | No | No MA | No MA | Yes | No | No MA | No |
| Silva *et al.*  2017 (61) | Acta Médica Portuguesa | Critically Low quality review | Yes | Partial yes | No | Partial yes | No | No | Yes | No | No | No | No MA | No MA | No | Yes | No MA | Yes |
| Simonelli *et al.*  2019 (62) | Pulmonology | Low quality review | Yes | No | Yes | Partial yes | No | Yes | Yes | Yes | Yes | No | No MA | No MA | Yes | Yes | No MA | Yes |
| Soares *et al.*  2011 (63) | Acta Médica Portuguesa | Critically Low quality review | Yes | No | No | Partial yes | No | No | Partial yes | No | No | No | No MA | No MA | No | No | No MA | Yes |
| Sousa *et al.*  2017 (64) | Acta Reumatológica Portuguesa | Low quality review | Yes | No | No | Partial yes | Yes | Yes | Partial yes | No | Yes | No | No MA | No MA | Yes | No | No MA | No |
| Tavares *et al.*  2013 (65) | Acta Médica Portuguesa | Critically Low quality review | Yes | No | No | Partial yes | Yes | Yes | No | Partial yes | No | No | No MA | No MA | No | No | No MA | Yes |
| Tinoco *et al.*  2018 (66) | Acta Médica Portuguesa | Low quality review | Yes | No | Yes | Partial yes | No | No | Partial yes | Partial yes | Yes | No | No MA | No MA | Yes | No | No MA | Yes |
| Trancas *et al.*  2011 (67) | Acta Médica Portuguesa | Critically Low quality review | No | Partial yes | No | Partial yes | No | No | No | No | No | No | No MA | No MA | No | No | No MA | Yes |
| Travensolo *et al. **  2018 (68) | Revista Portuguesa de Cardiologia | Low quality review | Yes | No | Yes | Partial yes | No | Yes | Yes | Partial yes | Yes | No | Yes | Yes | Yes | Yes | Yes | No |
| Trindade *et al.*  2015 (69) | Acta Médica Portuguesa | Critically Low quality review | Yes | Partial yes | No | Partial yes | No | No | No | Partial yes | No | No | No MA | No MA | No | No | No MA | Yes |
| Vargas *et al.*  2014 (70) | Acta Reumatológica Portuguesa | Critically Low quality review | Yes | No | No | Partial yes | Yes | No | Partial yes | Partial yes | No | No | No MA | No MA | No | Yes | No MA | No |
| Xu *et al.**  2017 (71) | Pulmonology | Critically Low quality review | Yes | No | No | Partial yes | Yes | No | Partial yes | Partial yes | No | No | Yes | No | No | Yes | Yes | Yes |

MA, Meta-analysis.

*The authors conducted a meta-analysis. †AMSTAR 2 Critical items.

Supplementary table 2 – Search Strategy

| **#** | **Searches using PubMed database** |
| --- | --- |
| 1 | meta-analysis OR meta-analyses OR “systematic review” OR “systematic reviews” |
| 2 | (((systematic review[ti] OR systematic literature review[ti] OR systematic scoping review[ti] OR systematic narrative review[ti] OR systematic qualitative review[ti] OR systematic evidence review[ti] OR systematic quantitative review[ti] OR systematic meta-review[ti] OR systematic critical review[ti] OR systematic mixed studies review[ti] OR systematic mapping review[ti] OR systematic cochrane review[ti] OR systematic search and review[ti] OR systematic integrative review[ti]) NOT comment[pt] NOT (protocol[ti] OR protocols[ti])) NOT MEDLINE [subset]) OR (Cochrane Database Syst Rev[ta] AND review[pt]) OR systematic review[pt] |
| 3 | "Acta medica portuguesa"[Jour] OR "Acta reumatologica portuguesa"[Jour] OR "Revista portuguesa de pneumologia"[Jour] OR “Pulmonology” [Jour] OR "Revista portuguesa de cirurgia cardio-toracica e vascular : orgao oficial da Sociedade Portuguesa de Cirurgia Cardio-Toracica e Vascular"[Jour] OR "Revista portuguesa de cardiologia : orgao oficial da Sociedade Portuguesa de Cardiologia = Portuguese journal of cardiology : an official journal of the Portuguese Society of Cardiology"[Jour] |
| 4 | (#1 OR #2) AND #3 |

Supplementary table 3 - General characteristics and PICO framework (PRISMA 2020^(72)^) of SRs included

| **Author, Publication Year** | **Portuguese Medical Journal** | **Number of studies included** | **RCT included** | **SR registration** | **Adherence to PRISMA guidelines*** | **Number of databases searched** | **Population** | **Intervention** | **Comparator** | **Outcome** |
| --- | --- | --- | --- | --- | --- | --- | --- | --- | --- | --- |
| AbuNurah *et al.*  2020 (1) | Pulmonology | 7 | No | Yes | No | 2 | Mechanically ventilated patients | NA | NA | NA |
| Alarcão *et al.*  2013 (2) | Acta Médica Portuguesa | 25 | Yes | No | No | 2 | Patients with Age-related macular degeneration, diabetic macular edema, branch retinal vein occlusion or central retinal vein occlusion | Intravitreal administration of bevacizumab or ranibizumab | Placebo or active comparator | NR |
| Albuquerque Baltar *et al.*  2010 (3) | Pulmonology | 4 | No | No | No | 3 | All patients with asthma | NA | NA | NA |
| Almeida *et al.*  2020 (4) | Acta Médica Portuguesa | 15 | No | No | No | 8 | Patients with intolerance to statins | NA | NA | NA |
| Andrade da Cunha *et al.*  2010 (5) | Pulmonology | 17 | No | No | No | 3 | Children with asthma | NA | NA | NA |
| Andrade *et al.*  2017 (6) | Acta Médica Portuguesa | 5 | No | Yes | Yes | 4 | Adults and older patients’ victims of a cerebrovascular event (preferably a primary event) | Speech Therapy | NR | Effects of speech therapy on dysphagia |
| Bogas *et al.*  2011 (7) | Acta Reumatológica Portuguesa | 65 | NR | No | No | 1 | Pregnant women and their foetuses | Biologic agents: infliximab, adalimumab, abatacept, rituximab, tocilizumab, golimumab, certolizumab, etanercept, anakira | NA | Pregnancy length, health condition of live births, neonatal complications, fetal development, congenital defects/malformations, miscarriages or elective terminations. |
| Branco *et al.*  2009 (8) | Acta Reumatológica Portuguesa | NR | NR | No | No | NR | NR | NR | NR | NR |
| Caldeira *et al.*†  2014 (9) | Revista Portuguesa de Cardiologia | 7 | No | No | No | 2 | All patients with atrial fibrilation or atrial flutter, regardless of its duration | Oral anticoagulation | NA | NA |
| Caldeira *et al.*†  2014a (10) | Revista Portuguesa de Cardiologia | 4 | Yes | No | No | 2 | Patients with chronic cardiac condition | Chronic cardiac medication | Chronic cardiac medication in a different regimen | Non-adherence to therapy |
| Campanilho-Marques *et al.*  2014 (11) | Acta Reumatológica Portuguesa | 9 | No | No | No | NR | Juvenile idiopathic arthritis (JIA)-associated uveitis | NA | NA | Prognostic role of antinuclear antibodies (ANA) |
| Carmona *et al.*  2007 (12) | Acta Reumatológica Portuguesa | 33 | Yes | No | No | 2 | NR | Any switch between biologic agents | NR | Any measure of efficacy, effectiveness, or safety from switching between biologic agents |
| Cavaca *et al.*  2017 (13) | Revista Portuguesa de Cardiologia | 57 | Yes | No | No | 1 | Patients with paradoxical aortic stenosis | NA | NA | NA |
| Cavalcante *et al.*†  2019 (14) | Revista Portuguesa de Cardiologia | 6 | Yes | No | Yes | 4 | All patients with asthma | NA | NA | Prognostic role of Maximal Inspiratory Pressure |
| Cavalcante Marcelino *et al.*  2010 (15) | Pulmonology | 26 | NR | No | No | 3 | Patients with cardiovascular disease | Structured program of exercise training | Standard medical care | Effectiveness of exercise training in increasing the number of circulating endothelial progenitor cells |
| Coelho *et al.*  2010 (16) | Acta Médica Portuguesa | NR | NR | No | No | 3 | Children with Attention Deficit Hyperactivity Disorder | NR | NR | NR |
| Costa *et al.*  2003 (17-19) | Revista Portuguesa de Cardiologia | 199 | Yes | No | No | NR | Patients with tricyclic intoxication | NA | NA | NA |
| Costa *et al.*  2001 (20-23) | Revista Portuguesa de Cardiologia | 34 | NR | No | No | 2 | Individuals with familiar hypercholesterolemia | NA | NA | NA |
| Costa *et al.*  2018a (24) | Acta Reumatológica Portuguesa | 7 | No | No | Yes | 2 | Patients with lumbar disc herniation with low back pain | Ozone therapy | Non-ozone therapy | Effectiveness and safety of ozone therapy |
| Costa *et al.*  2018 (25) | Acta Médica Portuguesa | 6 | Yes | Yes | Yes | 5 | Patients older than 18 years, with knee osteoarthritis | Intra-articular ozone therapy | Any pharmacologic or non-pharmacologic comparator | Improvement in pain based on the Visual Analogue Scale, evaluated at 1, 3, 6, and 12 months |
| Couto *et al.*†  2014 (26) | Acta Médica Portuguesa | 63 | NR | No | No | 1 | Patients with Parkinson’s disease | Deep brain stimulation | NR | Anxiety and depressive symptoms |
| Dores *et al.*  2013 (27) | Acta Médica Portuguesa | 288 | NR | No | No | 1 | NR | Virtual reality | NA | NA |
| Fabiao *et al.*  2010 (28) | Acta Médica Portuguesa | 12 | No | No | No | 1 | Patients with somatoform disorder | NA | NA | NA |
| Feitosa *et al.*  2012 (29) | Pulmonology | 6 | No | No | No | 4 | Patients with exercise induced bronchospasm | Exhaled nitric oxide | NA | NA |
| Ferreira *et al.*  2015 (30) | Revista Portuguesa de Cardiologia | 27 | No | No | No | 5 | Patients with non-valvular atrial fibrillation | Novel oral anticoagulants | Current standard of care | Cost effectiveness of the intervention |
| Ferreira *et al.*  2011 (31) | Acta Médica Portuguesa | 19 | No | No | No | 4 | Female patients (>18years) with stress urinary incontinence | Pelvic floor muscle training or pelvic floor muscle training together with other adjunctive therapies | Placebo, non-treatment, standard therapy | PFMT efficacy |
| Ferreira *et al.*†  2019 (32) | Acta Reumatológica Portuguesa | 39 | Yes | No | Yes | 8 | Patients with knee osteoarthritis | Non-surgical and nonpharmacological interventions | Pharmacological, surgical, placebo, no intervention, or other non-pharmacological/non-surgical interventions | Pain, physical function and patient global assessment |
| Fölter *et al.*  2011 (33) | Acta Reumatológica Portuguesa | 6 | NR | No | No | 2 | Women and/or men who were not being treated with drugs that altered bone quality and without other comorbidities that altered the bone mass index | NA | NA | NA |
| Francica *et al.*  2014 (34) | Acta Médica Portuguesa | 15 | Yes | No | No | 2 | Adults with a clinical diagnosis of stroke | Any aerobic physical training | NR | NR |
| Gomes Dos Reis Pimentel *et al.*  2017 (35) | Pulmonology | 11 | No | No | Yes^††^ | 1 | Participants diagnosed with IgE-mediated allergic respiratory disease, confirmed by objective measures (positive skin prick test and/or serum-specific IgE to sensitizing allergens) | Rush or cluster subcutaneous immunotherapy | Comparative intervention: placebo, conventional subcutaneous immunotherapy, or pharmacotherapy | To assess clinical and immunological efficacy as well as safety of accelerated SCIT build-up schedules for the treatment of respiratory allergy in pediatric patients |
| Gouveia *et al.*  2018 (36) | Acta Médica Portuguesa | 11 | Yes | No | No | 4 | Postmenopausal women, (physiological or surgical) with sexual dysfunction, quantified in a questionnaire | Testosterone, administered in any form, isolated or in association with estrogen and/or progesterone | Another hormonal (estrogen or progesterone) or non-hormonal (lubricant only) therapy | Improvement in the complaints of sexual dysfunction, quantified by a questionnaire |
| Guedes *et al.*  2014 (37) | Revista Portuguesa de Cardiologia | 5 | Yes | No | No | 6 | Patients with hyperuricemia | NA | NA | Reduction of cardiovascular events |
| Guerreiro *et al.*  2011 (38) | Acta Médica Portuguesa | NR | NR | No | No | 3 | Adolescents and young adults | Methylenedioxymethamphetamine, Methamphetamine, Lysergic Acid Diethylamide, Ketamine, Gamma-hydroxybutyrate, Flunitrazepam | NR | NR |
| Ladeiras-Lopes *et al.*†  2019 (39) | Revista Portuguesa de Cardiologia | 19 | No | No | Yes | 1 | Adults from the community that assessed diastolic function parameters | NA | NA | Association between Diastolic dysfunction and the incidence of major adverse cardiovascular events (MACE) and death |
| Liz Almeida *et al.*  2015 (40) | Revista Portuguesa de Cardiologia | 36 | NR | No | No | 1 | Individuals with implantable cardioverter-  defibrillators who participate in sports (athletes and non-athletes) | NA | NA | NA |
| Lopes *et al.*†  2014 (42) | Revista Portuguesa de Cardiologia | 19 | NR | No | No | 3 | Heart failure patients (NYHA class II-IV with left ventricular ejection fraction ≤35%) with atrial fibrillation and in sinus rhythm | Cardiac resynchronization therapy | No cardiac resynchronization therapy | All-cause mortality, cardiovascular mortality, non-responders to therapy and follow-up ≥6 months |
| Lopes *et al.*  2016 (41) | Acta Reumatológica Portuguesa | 8 | Yes | No | No | 1 | Patients with ankylosing spondylitis | Group exercise plans | NR | Scales such as Bath Ankylosing Spondylitis scales, Bath Ankylosing Spondylitis Metrology Index, Bath Ankylosing Spondylitis Functional Index, Bath Ankylosing Spondylitis Disease Activity Index, Health Assessment Questionnaire-Spondyloarthropathies, Medical Outcome Study Short Form 36 Healthy Survey-SF-36, Beck Depression Inventory |
| Lopes-Conceição *et al.*  2014 (43) | Revista Portuguesa de Cardiologia | 41 | NR | No | No | 1 | Patients with acute  coronary syndrome, stable angina and heart failure treated with pharmacological and non-pharmacological therapies in Portugal | NA | NA | NA |
| Mancuso *et al.*  2006 (44) | Pulmonology | 30 | NR | No | No | 3 | Patients of any age undergoing bone marrow transplantation | Pulmonary function test | NR | Pulmonary function testing: Total lung capacity, Forced expiratory volume e Carbon dioxide diffusion rate |
| Mancuzo *et al.*  2010 (45) | Pulmonology | 30 | No | No | No | 6 | NA | NA | NA | NA |
| Marinho-Dias *et al.*  2013 (46) | Acta Médica Portuguesa | 26 | No | No | No | 1 | Female patients undergoing pap smear | NA | NA | NA |
| Martins *et al.*†  2014 (47) | Acta Reumatológica Portuguesa | 19 | Yes | No | Yes | 4 | Patients with Ankylosing Spondylitis | Exercise | Physical activity | Functional impairment |
| Melo da Silva *et al.*†  2015 (48) | Revista Portuguesa de Cirurgia Cardio-torácica e Vascular | 23 | No | No | No | 2 | Newborns with a congenital cyanotic heart disease that is dependent of a patent ductus arteriosum | Percutaneous implantation of stent in duct procedure | modified Blalock-Taussig shunt | Primary outcome was overall mortality, secondary outcomes were procedure complications and need for re-intervention |
| Monjardino *et al.*  2011 (49) | Acta Reumatológica Portuguesa | 32 | No | No | Yes | 2 | Patients with rheumatic diseases | NA | NA | NA |
| Pacheco de Oliveira *et al.*  2015 (50) | Revista Portuguesa de Cirurgia Cardio-torácica e Vascular | 27 | No | No | Yes | 1 | Patients undergoing endovascular aortic repair, with evidence of internal iliac artery embolization | NA | NA | NA |
| Padilha J.M.  2010 (51) | Pulmonology | 4 | No | No | No | 9 | Patients with chronic obstructive pulmonary disease | NA | NA | Promotion of self-management |
| Paneroni *et al.*†  2020 (52) | Pulmonology | 16 | Yes | No | Yes | 4 | patients with chronic obstructive pulmonary disease | Exercise training programs | Usual care or education | Impact of exercise training on fatigue |
| Peixoto *et al.*  2013 (53) | Acta Reumatológica Portuguesa | 13 | No | No | No | 3 | Patients with idiopathic inflammatory myopathies | NA | NA | Potential of myositis-specific antibodies (MSAs) in the disease course and clinical characteristics |
| Pereira Da Silva *et al.*  2010 (54) | Acta Médica Portuguesa | 28 | NR | No | No | 3 | Newborn infants | NA | NA | NA |
| Pinheiro *et al.*†  2019 (55) | Acta Médica Portuguesa | 10 | No | No | Yes | 3 | Women of advanced maternal age | NA | NA | Obstetrical and perinatal outcomes |
| Pisani *et al.*  2019 (56) | Pulmonology | 5 | Yes | No | No | 4 | Patients with exacerbated chronic obstructive pulmonary disease | High-Flow through Nasal Cannula | Oxygen therapy or non-invasive ventilation | Effects of High-Flow through Nasal Cannula use in exacerbated chronic obstructive pulmonary disease patients |
| Quintas-Neves *et al.*  2016 (57) | Pulmonology | 22 | Yes | No | No | 1 | Patients with obstructive sleep apnea undergoing bariatric surgery | Bariatric surgery | Other types of bariatric surgery | Improvement in OSA symptoms, measured with Epworth Sleeping Scale; Berlin Questionnaire; Pittsburgh Sleep Quality Index; Technion Sleep Questionnaire |
| Raposo *et al.*  2016 (58) | Acta Reumatológica Portuguesa | 27 | No | No | Yes | 1 | Patients with rheumatoid arthritis, Sjögren’s syndrome, systemic lupus erythematosus and systemic sclerosis | NA | NA | Association between human papillomavirus infection and cervical lesions in rheumatic diseases |
| Ribeiro *et al.*  2010 (59) | Acta Reumatológica Portuguesa | 267 | No | No | No | 3 | Patients with undifferentiated peripheral inflammatory arthritis | NA | NA | NA |
| Ribeiro *et al.*  2007 (60) | Acta Reumatológica Portuguesa | 13 | Yes | No | No | 3 | Patients with ankylosing spondylitis (AS) | Physical exercise | Other exercise-based approaches, Pharmacological, no intervention | Prevention and management of the deformities related to AS |
| Silva *et al.*  2017 (61) | Acta Médica Portuguesa | 14 | No | No | Yes | 3 | Patients older than 65 years old, who had undergone surgical repair of a symptomatic rotator cuff tear | Surgical Treatment | NR | Efficacy of surgical treatment |
| Simonelli *et al.*  2019 (62) | Pulmonology | 6 | Yes | No | Yes | 4 | Patients with chronic obstructive pulmonary disease | Manual therapy (alone or added to exercise) | Usual care, to sham techniques or light manual interventions, physical activity, exercise alone | Effectiveness of manual therapy |
| Soares *et al.*  2011 (63) | Acta Médica Portuguesa | 10 | No | No | No | 2 | Patient ≥65 years | Prescription of potentially inappropriate medication | NA | NA |
| Sousa *et al.*  2017 (64) | Acta Reumatológica Portuguesa | 28 | Yes | No | No | 2 | Patients with systemic inflammatory rheumatic diseases, aged < 18 | Vaccination | vaccinated healthy children or children with SIRD that were randomly not vaccinated; | efficacy of the immunization defined as seroconversion (≥4-fold increase in post vaccination titer), seroprotection (antibody titers ≥10 mIU/ml in case of hepatitis B virus vaccine or ≥40 IU/ml after immunization in all other vaccines) or increase in the serum geometric mean titers (GMT) of antibodies; and the safety and tolerability of vaccination estimated by flares of rheumatic disease, local and systemic adverse events related to the administration of vaccines and episodes of overt disease like varicella or rubella |
| Tavares *et al.*  2013 (65) | Acta Médica Portuguesa | 41 | NR | No | No | 2 | Advanced cancer patients (≥18 years) | NA | NA | Prognosis in advanced cancer patients |
| Tinoco *et al.*  2018 (66) | Acta Médica Portuguesa | 7 | Yes | No | No | 7 | Patients with recurrent urinary tract infection | Uro-Vaxom® | Placebo or pre-treatment | Decrease of lower urinary tract infections recurrences |
| Trancas *et al.*  2011 (67) | Acta Médica Portuguesa | 28 | NR | No | No | 1 | Forensic psychiatry residents | NA | NA | NA |
| Travensolo *et al.*†  2018 (68) | Revista Portuguesa de Cardiologia | 15 | Yes | No | No | 2 | Patients with cardiovascular disease | NA | NA | NA |
| Trindade *et al.*  2015 (69) | Acta Médica Portuguesa | 19 | Yes | No | Yes | 3 | Patients with musculoskeletal diseases | Periarticular corticosteroid injection | NR | Safety and effectiveness |
| Vargas *et al.*  2014 (70) | Acta Reumatológica Portuguesa | 12 | No | No | No | 2 | Systemic Lupus Erythematosus Patients | NA | NA | NA |
| Xu *et al.*†  2017 (71) | Pulmonology | 9 | No | No | No | 6 | NR | NA | NA | Correlation between leptin receptor gene polymorphisms and the risk of obstructive sleep apnea syndrome |

PRISMA, Preferred Reporting Items for Systematic Reviews and Meta-Analyses; RCT, Randomized controlled trial; SRs, Systematic reviews; NR, Not reported; NA, Not applicable according to Munn *et alI.*’s.^(73)^ question format (Reviews’ questions aligned with the 10 review types).
*Authors report they followed Preferred Reporting Items for Systematic Reviews and Meta-Analyses (PRISMA) guidelines. † The authors conducted a meta-analysis. †† The authors followed Centre for Reviews and Dissemination's (CRD) guidance for undertaking reviews in health care.

**References**

1. AbuNurah HY, Russell DW, Lowman JD. The validity of surface EMG of extra-diaphragmatic muscles in assessing respiratory responses during mechanical ventilation: A systematic review. Pulmonology. 2020;26(6):378-85.

2. Alarcão J, Costa J, Fareleira F, Borges M, Carneiro AV. [Off-label prescribing: scientific analysis taking the use of bevacizumab in ophthalmology as an example]. Acta Med Port. 2013;26(4):409-19.

3. Albuquerque Baltar J, Brasileiro Santos Mdo S, Justino da Silva H. [Does asthma promote changes in static posture? - Systematic review]. Rev Port Pneumol. 2010;16(3):471-6.

4. Almeida JT, Esteves AL, Martins F, Palma I. [Approach to Patients with Statin Intolerance: Evidence-Based Review]. Acta Med Port. 2020;33(1):49-57.

5. Andrade da Cunha D, Justino da Silva H, Pernambuco Lde A, Juliana RdMK, Jeronimo do Prado I, Moura de Andrade G, et al. [Effects of asthma on nutritional status in children: a systematic review]. Rev Port Pneumol. 2010;16(4):617-26.

6. Andrade JS, Souza W, Paranhos LR, Domenis DR, César C. [Effects of Speech Therapy in Hospitalized Patients with Post-Stroke Dysphagia: A Systematic Review of Observational Studies]. Acta Med Port. 2017;30(12):870-81.

7. Bogas M, Leandro MJ. Biologic therapy and pregnancy. A systematic literature review. Acta Reumatol Port. 2011;36(3):219-32.

8. Branco JC, Felicíssimo P, Monteiro J. [Epidemiology of hip fractures and its social and economic impact. A revision of severe osteoporosis current standard of care]. Acta Reumatol Port. 2009;34(3):475-85.

9. Caldeira D, Barra M, David C, Costa J, Ferreira JJ, Pinto FJ. The prevalence of oral anticoagulation in patients with atrial fibrillation in Portugal: Systematic review and meta-analysis of observational studies. Rev Port Cardiol. 2014;33(9):555-60.

10. Caldeira D, Vaz-Carneiro A, Costa J. The impact of dosing frequency on medication adherence in chronic cardiovascular disease: systematic review and meta-analysis. Rev Port Cardiol. 2014;33(7-8):431-7.

11. Campanilho-Marques R, Bogas M, Ramos F, Santos MJ, Fonseca JE. Prognostic value of antinuclear antibodies in juvenile idiopathic arthritis and anterior uveitis. Results from a systematic literature review. Acta Reumatol Port. 2014;39(2):116-22.

12. Carmona L, Ortiz A, Abad MA. How good is to switch between biologics? A systematic review of the literature. Acta Reumatol Port. 2007;32(2):113-28.

13. Cavaca R, Teixeira R, Vieira MJ, Gonçalves L. Paradoxical aortic stenosis: A systematic review. Rev Port Cardiol. 2017;36(4):287-305.

14. Cavalcante SL, Lopes S, Bohn L, Cavero-Redondo I, Álvarez-Bueno C, Viamonte S, et al. Effects of exercise on endothelial progenitor cells in patients with cardiovascular disease: A systematic review and meta-analysis of randomized controlled trials. Revista Portuguesa de Cardiologia. 2019;38(11):817-27.

15. Cavalcante Marcelino AM, da Silva HJ. Role of maximal inspiratory presure in the evaluetion of respiratory muscle strength in asthmatics - Systematic review. Rev Port Pneumol. 2010;16(3):463-70.

16. Coelho L, Chaves E, Vasconcelos S, Fonteles M, De Sousa F, Viana G. [Attention deficit hyperactivity disorder (ADHD) in children: neurobiological aspects, diagnosis and therapeutic approach]. Acta Med Port. 2010;23(4):689-96.

17. Costa J, Borges M, Oliveira E, Gouveia M, Carneiro AV. Incidence and prevalence of hypercholesterolemia in Portugal: a systematic review. Part III. Rev Port Cardiol. 2003;22(6):829-36.

18. Costa J, Borges M, Oliveira E, Gouveia M, Carneiro AV. Incidence and prevalence of hypercholesterolemia in Portugal: a systematic review. Part I. Rev Port Cardiol. 2003;22(4):569-77.

19. Costa J, Borges M, Oliveira E, Gouveia M, Carneiro AV. Incidence and prevalence of hypercholesterolemia in Portugal: a systemic review. Part II. Rev Port Cardiol. 2003;22(5):683-702.

20. Costa J, de Oliveira EI, Rosa MM, Ferreira JJ, Sampaio C, Carneiro AV. [Cardiac effects of acute poisoning with tricyclic antidepressants: systematic review of the literature -- Part IV]. Rev Port Cardiol. 2001;20(10):1021-30.

21. Costa J, de Oliveira EI, Rosa MM, Ferreira JJ, Sampaio C, Carneiro AV. [Cardiac effects of acute poisoning with tricyclic antidepressants: systematic review of the literature -- Part II]. Rev Port Cardiol. 2001;20(7-8):765-72.

22. Costa J, de Oliveira EI, Rosa MM, Ferreira JJ, Sampaio C, Carneiro AV. [Cardiac effects of acute poisoning with tricyclic antidepressants: systematic review of the literature -- Part III]. Rev Port Cardiol. 2001;20(9):901-9.

23. Costa J, Rosa MM, Ferreira JJ, Sampaio C, Vaz Carneiro A. [Cardiac effects of acute poisoning with tricyclic antidepressants: systematic review of the literature. Part I]. Rev Port Cardiol. 2001;20(6):671-8.

24. Costa T, Rodrigues-Manica S, Lopes C, Gomes J, Marona J, Falcão S, et al. [Ozone Therapy in Knee Osteoarthritis: A Systematic Review]. Acta Med Port. 2018;31(10):576-80.

25. Costa T, Linhares D, Ribeiro da Silva M, Neves N. Ozone therapy for low back pain. A systematic review. Acta Reumatol Port. 2018;43(3):172-81.

26. Couto MI, Monteiro A, Oliveira A, Lunet N, Massano J. Depression and anxiety following deep brain stimulation in Parkinson's disease: systematic review and meta-analysis. Acta Med Port. 2014;27(3):372-82.

27. Dores AR, Barbosa F, Marques A, Carvalho IP, De Sousa L, Castro-Caldas A. [Virtual reality and rehabilitation: why or why not? A systematic literature review]. Acta Med Port. 2012;25(6):414-21.

28. Fabião C, Silva MC, Fleming M, Barbosa A. [Somatoform disorders - a revision of the epidemiology in primary health care]. Acta Med Port. 2010;23(5):865-72.

29. Feitosa LA, Dornelas de Andrade A, Reinaux CM, Britto MC. Diagnostic accuracy of exhaled nitric oxide in exercise-induced bronchospasm: Systematic review. Rev Port Pneumol. 2012;18(4):198-204.

30. Ferreira J, Mirco A. Systematic review of cost-effectiveness analyses of novel oral anticoagulants for stroke prevention in atrial fibrillation. Rev Port Cardiol. 2015;34(3):179-91.

31. Ferreira M, Santos P. [Pelvic floor muscle training programmes: a systematic review]. Acta Med Port. 2011;24(2):309-18.

32. Ferreira RM, Torres RT, Duarte JA, Gonçalves RS. Non-Pharmacological and Non-Surgical Interventions for Knee Osteoarthritis: A Systematic Review and Meta-Analysis. Acta Reumatol Port. 2019;44(3):173-217.

33. Flöter M, Bittar CK, Zabeu JL, Carneiro AC. Review of comparative studies between bone densitometry and quantitative ultrasound of the calcaneus in osteoporosis. Acta Reumatol Port. 2011;36(4):327-35.

34. Francica JV, Bigongiari A, Mochizuki L, Miranda ML, Rodrigues B. Aerobic program in persons with stroke: a systematic review. Acta Med Port. 2014;27(1):108-15.

35. Gomes Dos Reis Pimentel RA, Oliveira G, Ferreira Chaves Loureiro ELCS. Accelerated subcutaneous immunotherapy in pediatric population - Systematic review. Pulmonology. 2017.

36. Gouveia M, Sanches R, Andrade S, Carmona S, Ferreira C. [The Role of Testosterone in The Improvement of Sexual Desire in Postmenopausal Women: An Evidence-Based Clinical Review]. Acta Med Port. 2018;31(11):680-90.

37. Guedes M, Esperança A, Pereira AC, Rego C. What is the effect on cardiovascular events of reducing hyperuricemia with allopurinol? An evidence-based review. Rev Port Cardiol. 2014;33(11):727-32.

38. Guerreiro DF, Carmo AL, da Silva JA, Navarro R, Góis C. [Club drugs]. Acta Med Port. 2011;24(5):739-56.

39. Ladeiras-Lopes R, Araújo M, Sampaio F, Leite-Moreira A, Fontes-Carvalho R. The impact of diastolic dysfunction as a predictor of cardiovascular events: A systematic review and meta-analysis. Revista Portuguesa de Cardiologia. 2019;38(11):789-804.

40. Liz Almeida R, Providência R, Gonçalves L. Use of implantable cardioverter-defibrillators in athletes: A systematic review. Rev Port Cardiol. 2015;34(6):411-9.

41. Lopes S, Costa S, Mesquita C, Duarte J. [Home based and group based exercise programs in patients with ankylosing spondylitis: systematic review]. Acta Reumatol Port. 2016;41(2):104-11.

42. Lopes C, Pereira T, Barra S. Cardiac resynchronization therapy in patients with atrial fibrillation: a meta-analysis. Rev Port Cardiol. 2014;33(11):717-25.

43. Lopes-Conceição L, Pereira M, Araújo C, Laszczýnska O, Lunet N, Azevedo A. The use of reperfusion and revascularization procedures in acute coronary syndrome in Portugal: a systematic review. Rev Port Cardiol. 2014;33(11):707-15.

44. Mancuso EV, Rezende NA. [Pulmonary function testing in bone marrow transplantation: a systematic review]. Rev Port Pneumol. 2006;12(1):61-9.

45. Mancuzo EV, Neves MA, Bittencourt H, de Rezende NA. [Non-infectious pulmonary complications after the hematopoietic stem cell transplantation]. Rev Port Pneumol. 2010;16(5):815-28.

46. Marinho-Dias J, Sousa H. Cytomegalovirus infection and cervical cancer: from past doubts to present questions. Acta Med Port. 2013;26(2):154-60.

47. Martins NA, Furtado GE, Campos MJ, Leitão JC, Filaire E, Ferreira JP. Exercise and ankylosing spondylitis with New York modified criteria: a systematic review of controlled trials with meta-analysis. Acta Reumatol Port. 2014;39(4):298-308.

48. Melo da Silva E, Casanova J. [Cyanotic congenital heart diseases duct dependent: Stent versus surgical shunt. Meta-analysis of observational studies]. Rev Port Cir Cardiotorac Vasc. 2015;22(3):135-44.

49. Monjardino T, Lucas R, Barros H. Frequency of rheumatic diseases in Portugal: a systematic review. Acta Reumatol Port. 2011;36(4):336-63.

50. Pacheco de Oliveira JC, Santos JV, Moreira Sampaio SM. [Complications of Hypogastric Arteries Embolization in EVAR]. Rev Port Cir Cardiotorac Vasc. 2015;22(1):41-6.

51. Padilha JM. [Influence of perception of chronic obstructive pulmonary disease in promotion of self-management of disease]. Rev Port Pneumol. 2010;16(4):641-8.

52. Paneroni M, Vitacca M, Venturelli M, Simonelli C, Bertacchini L, Scalvini S, et al. The impact of exercise training on fatigue in patients with chronic obstructive pulmonary disease: a systematic review and meta-analysis. Pulmonology. 2020;26(5):304-13.

53. Peixoto D, Costa J, Ferretti M, Malattia C, Martini A. New autoantibodies and their clinical associations in juvenile myositis - a systematic review. Acta Reumatol Port. 2013;38(4):234-41.

54. Pereira Da Silva T, Justo Da Silva L. [Pain scales used in the newborn infant: a systematic review]. Acta Med Port. 2010;23(3):437-54.

55. Pinheiro RL, Areia AL, Mota Pinto A, Donato H. Advanced Maternal Age: Adverse Outcomes of Pregnancy, A Meta-Analysis. Acta Med Port. 2019;32(3):219-26.

56. Pisani L, Astuto M, Prediletto I, Longhini F. High flow through nasal cannula in exacerbated COPD patients: a systematic review. Pulmonology. 2019;25(6):348-54.

57. Quintas-Neves M, Preto J, Drummond M. Assessment of bariatric surgery efficacy on Obstructive Sleep Apnea (OSA). Rev Port Pneumol (2006). 2016;22(6):331-6.

58. Raposo A, Tani C, Costa J, Mosca M. Human papillomavirus infection and cervical lesions in rheumatic diseases: a systematic review. Acta Reumatol Port. 2016;41(3):184-90.

59. Ribeiro A, Machado P, Ramiro S, Duarte C, Mourão AF, Bogas M, et al. [Portuguese recommendations on "How to diagnose, monitor, and treat undifferentiated peripheral arthritis": systematic review of the literature and the experts' opinion]. Acta Reumatol Port. 2010;35(5):475-96.

60. Ribeiro F, Leite M, Silva F, Sousa O. [Physical exercise in the treatment of Ankylosing Spondylitis: a systematic review]. Acta Reumatol Port. 2007;32(2):129-37.

61. Silva BM, Cartucho A, Sarmento M, Moura N. Surgical Treatment of Rotator Cuff Tears After 65 Years of Age: A Systematic Review. Acta Med Port. 2017;30(4):320-9.

62. Simonelli C, Vitacca M, Vignoni M, Ambrosino N, Paneroni M. Effectiveness of manual therapy in COPD: A systematic review of randomised controlled trials. Pulmonology. 2019;25(4):236-47.

63. Soares MA, Fernandez-Llimos F, Cabrita J, Morais J. [Tools to evaluate potentially inappropriate prescription in the elderly: a systematic review]. Acta Med Port. 2011;24(5):775-84.

64. Sousa S, Duarte AC, Cordeiro I, Ferreira J, Gonçalves MJ, Meirinhos T, et al. Efficacy and Safety of Vaccination in Pediatric Patients with Systemic Inflammatory Rheumatic Diseases: a systematic review of the literature. Acta Reumatol Port. 2017;42(1):8-16.

65. Tavares T, Gonçalves E. [Vital prognosis in advanced cancer patients: a systematic literature review]. Acta Med Port. 2013;26(4):433-44.

66. Tinoco M, Santos M, Reitor C, Rodrigues A, Monteiro J. [Polysaccharide of Escherichia coli in the Prevention of Recurrent Urinary Tract Infection: An Evidence-Based Review]. Acta Med Port. 2018;31(3):165-9.

67. Trancas B, Vieira F, Costa Santos J. [Training in forensic psychiatry: comparative issues towards a rethinking of the Portuguese model]. Acta Med Port. 2011;24 Suppl 4:845-54.

68. Travensolo C, Goessler K, Poton R, Pinto RR, Polito MD. Measurement of physical performance by field tests in programs of cardiac rehabilitation: a systematic review and meta-analysis. Revista Portuguesa de Cardiologia (English Edition). 2018;37(6):525-37.

69. Trindade I. [Periarticular Corticosteroid Injection in the Therapeutic Approach of Musculoskeletal Disease in General Practice: A systematic Review]. Acta Med Port. 2015;28(5):652-62.

70. Vargas JV, Vaz CJ. Evaluation of central nervous system involvement in SLE patients. Screening psychiatric manifestations--a systematic review. Acta Reumatol Port. 2014;39(3):208-17.

71. Xu B, Liu J, Li T, Liu S. Gln223Arg polymorphism in the Caucasian population and Pro1019Pro polymorphism in the Chinese population are risk factors for OSAS: An updated meta-analysis of 1159 subjects. Rev Port Pneumol (2006). 2017;23(2):62-70.

72. Page MJ, McKenzie JE, Bossuyt PM, Boutron I, Hoffmann TC, Mulrow CD, et al. The PRISMA 2020 statement: an updated guideline for reporting systematic reviews. International Journal of Surgery. 2021;88:105906.

73. Munn Z, Stern C, Aromataris E, Lockwood C, Jordan Z. What kind of systematic review should I conduct? A proposed typology and guidance for systematic reviewers in the medical and health sciences. BMC Med Res Methodol. 2018;18(1):5.
